# Chronic Kidney Disease and Heart Failure–Everyday Diagnostic Challenges

**DOI:** 10.3390/diagnostics11112164

**Published:** 2021-11-22

**Authors:** Anna Adamska-Wełnicka, Marcin Wełnicki, Artur Mamcarz, Ryszard Gellert

**Affiliations:** 1 Clinic of Nephrology and Internal Medicine, Centre of Postgraduate Medical Education, 01-813 Warsaw, Poland; anna.adamska-welnicka@cmkp.edu.pl (A.A.-W.); rgellert@cmkp.edu.pl (R.G.); 2 3rd Department of Internal Medicine and Cardiology, Medical University of Warsaw, 02-091 Warsaw, Poland; artur.mamcarz@wum.edu.pl

**Keywords:** heart failure, chronic kidney disease, pulmonary hypertension, arteriovenous fistulas, overhydration

## Abstract

Is advanced chronic kidney disease (CKD) a cardiac “no man’s land”? Chronic heart failure (HF) is widely believed to be one of the most serious medical challenges of the 21st century. Moreover, the number of patients with CKD is increasing. To date, patients with estimated glomerular filtration rates <30 mL/min/1.73 m^2^ have frequently been excluded from large, randomized clinical trials. Although this situation is slowly changing, in everyday practice we continue to struggle with problems that are not clearly addressed in the guidelines. This literature review was conducted by an interdisciplinary group, which comprised a nephrologist, internal medicine specialists, and cardiologist. In this review, we discuss the difficulties in ruling out HF for patients with advanced CKD and issues regarding the cardiotoxicity of dialysis fistulas and the occurrence of pulmonary hypertension in patients with CKD. Due to the recent publication of the new HF guidelines by the European Society of Cardiology, this is a good time to address these difficult issues. Contrary to appearances, these are not niche issues, but problems that affect many patients.

## 1. Introduction

Chronic heart failure (HF) is one of the greatest medical challenges of the 21st century. It is estimated that HF currently affects 1–2% of the adult population in developed countries [1]. Moreover, the prevalence of HF rises significantly among people over 70, reaching 10% [2]. According to data from the European Society of Cardiology (ESC) Long-Term Registry, most outpatients with HF (60%) have a reduced left ventricle ejection fraction (LVEF), about one quarter (24%) have mildly reduced LVEF, and the remainder (16%) have preserved LVEF [3]. Chronic kidney disease (CKD) is also a very serious public health problem. The prevalence of CKD in the general population is estimated at about 9–16%, and it has increased by almost 30% over the last three decades [4]. HF and CKD frequently coexist; according to a meta-analysis by Damman et al., CKD is found in approximately half of patients with HF [5]. Similar observations were made by McAlister et al., who found features of kidney damage in 43% of patients with chronic HF and in 53% of patients with acute HF [6]. However, it would be difficult to determine how many patients with CKD have concurrent HF, because few studies report those specific numbers. In the guidelines for the diagnosis and treatment of HF, the section on CKD indicates that most studies conducted to date have used an estimated glomerulus filtration rate (eGFR) of <30 mL/min/1.73 m^2^ as a criterion for excluding patients with HF [1]. Therefore, advanced CKD is a kind of “no man’s land” for many of us in everyday clinical practice. Research has shown that the coexistence of HF and CKD doubles the risk of death. A sharp increase in mortality is observed when the eGFR value drops below 60 mL/min/1.73 m^2^, and the highest mortality rates are found among patients with HF and end stage kidney disease, i.e., when the eGFR is <15 mL/min/1.73 m^2^ [5,7]. The latter group also includes patients on dialysis. Therefore, there is no doubt that this population requires special attention. In practice, however, a patient with severe renal impairment presents a dual challenge: first, in the context of therapeutic decisions, and second, in the context of diagnosing HF. HF is described as a clinical syndrome consisting of major symptoms and signs: dyspnea, peripheral edema and pulmonary congestion. Echocardiography and plasma concentration of natriuretic peptides play a key role in HF diagnosis [1]. At the same time, overhydration in the course of advanced renal failure causes similar symptoms and signs, has an impact on the heart’s function and structure, and makes it difficult to interpret natriuretic peptide concentration [8]. With the recent publication of the new ESC guidelines on HF, this is a good time to focus on the most difficult and debated issues at the interface between cardiology and nephrology.

## 2. Materials and Methods

We searched PubMed for articles with the key words “heart failure”, “chronic kidney disease”, “pulmonary hypertension”, “arteriovenous fistula”, “natriuretic peptides”, “fluid overload”, “end stage renal disease”, and “hemodialysis”. In particular, we used the following search terms and logic: “chronic kidney disease AND heart failure” OR pulmonary hypertension AND “chronic kidney disease OR heart failure” or “chronic kidney disease AND arteriovenous fistula AND cardiotoxicity”. Additional studies were identified by examining the references of some articles. Articles were selected according to their title and abstract, based on eligibility criteria. We included English articles, adult populations, and all types of study, including narrative and systematic reviews, clinical studies, case reports, and expert opinions. Manuscripts published in a language other than English were excluded. In general, we excluded studies published earlier than in 2010, but we made single exceptions to this rule. The final analysis included 61 articles, which we selected, based on originality and relevance to the broader scope of our review. In the discussion of the basic issues concerning the pharmacotherapy of HF in the coexistence of advanced CKD, an additional 11 articles were cited, according to the subjective assessment of the authors of the review.

## 3. How to Confirm or Rule out HF in a Patient

**Figure 1:**
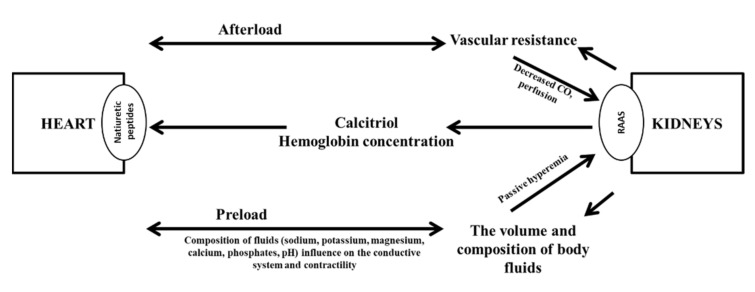
Basic mechanisms underlying the mutual dependencies of heart and kidney functions. CO, cardiac output; RAAS, renin-angiotensin-aldosterone system. A figure prepared by the authors on the basis of pathophysiological issues described by Rangaswami et al. [8]. The figure shows the influence of heart and kidney function on the afterload and preload values. The decrease in cardiac output due to heart failure causes renal hypoperfusion. The sympathetic nervous system, the renin-angiotensin-aldosterone system and the release of vasopressin are activated in order to increase systemic vascular resistance and circulating blood volume. Increased venous pressure causes renal congestion, which additionally impairs their function. A decrease in glomerular filtration causes not only hypervolemia (increased preload), but also unfavorable changes in ion concentrations, which affects the contractility of cardiomyocytes. In addition, along with the deteriorating kidney function, disturbances in calcium-phosphate metabolism increase and the synthesis of erythropoietin is impaired, which also adversely affects the function of blood vessels and the heart muscle.

According to the current diagnostic algorithm, we suspect HF on the basis of typical symptoms and signs in a patient with HF risk factors and an incorrect ECG [1]. A concentration of BNP ≥ 35 pg/mL or NT-proBNP ≥ 125 pg/mL and abnormal findings in echocardiography confirm the diagnosis. Echocardiography also allows determination of the HF phenotype [1]. HF and CKD share many risk factors. Among these, hypertension and diabetes are the most important. In practice, however, it is important to realize that HF can be both a cause and a consequence of renal failure. The basic pathophysiological mechanisms underlying this relationship are presented in Figure 1. To emphasize the frequent coexistence and close relationship between cardiovascular diseases and kidney diseases, the term “cardio–renal syndrome” was coined [8].

Nosologically, neither HF nor CKD are disease entities; instead, they are clinical syndromes. HF is currently defined as a “clinical syndrome consisting of cardinal symptoms (e.g., breathlessness, ankle swelling, and fatigue) that may be accompanied by signs (e.g., elevated jugular venous pressure, pulmonary crackles, and peripheral edema). HF is due to a structural and/or functional abnormality of the heart that results in elevated intracardiac pressures and/or inadequate cardiac output (CO) at rest and/or during exercise.” [1]. The definition of CKD has not changed for almost a decade. CKD is diagnosed, when, for at least 3 months, we find one or both of the following signs:

1. A reduction in the eGFR to < 60 mL/min/1.73 m^2^
2. Kidney damage in imaging, histopathology, or laboratory tests.

**Figure 2:**
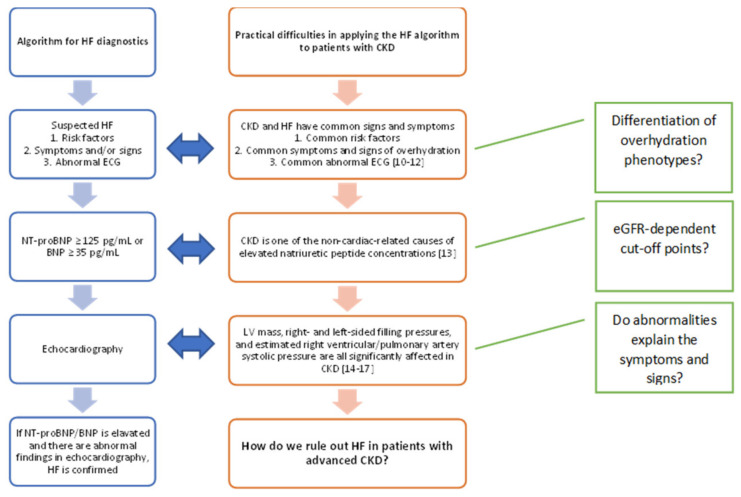
Difficulties in implementing the current diagnostic algorithm for HF for patients with CKD [1,10,11,12,13,14,15,16,17]. The HF diagnostic algorithm (left) includes symptoms that are common in patients with CKD, with or without HF (center). Ruling out HF in patients with CKD may require further differentiation; (right) some potential differentiation factors are proposed; For example, arterial hypertension and diabetes are the primary risk factors for both CKD and HF. In a patient with a significantly reduced eGFR, dyspnea and oedema may be caused by both CKD and concomitant or occurring de novo HF. With the deteriorating renal function, the unequivocal interpretation of the elevated concentration of natriuretic peptides becomes troublesome and electrolyte disturbances may affect the ECG. In such a setting, it is difficult to conclude whether HF coexists with CKD or whether the deterioration of cardiac function is secondary to renal failure and therefore potentially reversible. This issue is discussed in detail later in the article; HF: heart failure; CKD: chronic kidney disease; ECG: electrocardiogram; BNP: brain natriuretic peptide; LV: left ventricular; eGFR: estimated glomerular filtration rate.

Therefore, the diagnosis of CKD is determined by specific tests, not by clinical symptoms. According to the Kidney Disease: Improving Global Outcomes guidelines of 2012, there are five stages of CKD. A correct classification of the CKD stage requires assessments of GFR and albuminuria, and to determine the combination of causes; thus, the classification includes the GFR category (G1–G5), and albuminuria category (A1–A3), as proposed in the guidelines [9]. Hence, the diagnosis of CKD is straightforward in a patient with previously diagnosed HF. However, for patients with previously diagnosed CKD, the usefulness of the HF diagnostic algorithm currently proposed may be considered questionable [1] (Figure 2).

## 4. Is the Nature of Overhydration the Same in HF and CKD?

The circulatory system consists of four main compartments—venous, arterial, pulmonary, and systemic. The distribution of fluid volumes within these compartments is asymmetric. The arterial system comprises 20–30% and the venous system comprises 70–80% of the fluid volume. The structure and function of the heart contribute to the maintenance of this physiological asymmetry. When disorders in the heart’s pumping function outweigh disorders of the return function, the cardiac muscle cannot generate an adequate CO, due to low stroke volume. The result is called forward failure. Conversely, when the return dysfunction is predominant, the result is called backward failure, and the predominant venous outflow results in organ congestion. Therefore, the consequence of HF is a redistribution of the intravascular volume, which leads to a permanent underfilling of the arterial compartment (10–15%) and displacement of the "extra" volume into the venous compartment (85–95%). Thus, ultimately, in HF, fluid overload is most often associated with hypovolemia. However, it should be emphasized that, in patients with CKD, fluid overload is associated with hypervolemia. As shown in Figure 1, in HF, hypovolemia is one of the causes of kidney damage (types 1 and 2 of the cardio-renal syndrome) [8]. Conversely, hypervolemia in the course of renal failure can cause HF (types 3 and 4 of the cardio-renal syndrome) [18]. The classification of cardio-renal syndromes systematizes knowledge; however, it is not easily translated into everyday practice [8]. Indeed, in everyday practice, it is sufficient to identify the coexistence of HF and renal dysfunction and to identify the initiating factor. Then, the initiating factor is the target of the therapy. Moreover, patients may not present with only one specific type of cardio-renal syndrome; they can change between types and even present two types at the same time [8].

A physical examination generally assesses the patient’s signs and symptoms, and severity is graded with the New York Heart Association (NYHA) classification. The examination typically includes a blood pressure measurement and assessments of the presence and severity of peripheral edema and the detection of crackles over the pulmonary fields. However, these assessments are not sufficient to make a reliable assessment of the degree of fluid overload [19,20,21]. Moreover, it remains challenging to assess the correct hydration status in patients that require dialysis. Consequently, the medical examination results are objectified with additional methods; for example, the whole body electrical bioimpedance can be measured; ultrasound can be performed to assess inferior vena cava compliance; or ultrasound can be performed to assess pulmonary congestion [21]. Each of these methods has advantages and limitations (Table 1).

The use of additional assessment methods is time-consuming. Therefore, research is ongoing on the development of simplified diagnostic schemes. Among the biochemical markers for assessing hydration status, natriuretic peptide concentrations are often measured. Both brain natriuretic peptide (BNP) and the inactive N-terminal prohormone-BNP (NT-proBNP) are produced at high levels in HF. However, the results should be interpreted carefully.

## 5. What Is the Practical Significance of BNP and NT-proBNP in Patients with Advanced CKD?

Normally when HF is suspected, BNP/NT-proBNP concentration is of key importance in the diagnostic algorithm [1]. They are also good prognostic markers [1]. However, the coexistence of renal failure may influence the diagnostic value of BNP/NT-proBNP. It is believed that, for stages 1–2 CKD, standard BNP cut-off thresholds can be used to diagnose HF. In the more advanced stages of kidney damage, research shows that the cut-off point must be adjusted. The NT-proBNP concentration is more dependent on kidney function than the BNP concentration [22]. In patients with a similar degree of LV dysfunction, natriuretic peptide concentrations are significantly higher in the presence of renal failure, and they are positively correlated with a reduction in the GFR [22]. A study by Vickery et al. showed that, for each 10 mL/min reduction in GFR, there was a 38% increase in the NT-pro-BNP concentration [23]. 

For patients with stage 3–5 CKD, the BNP threshold for diagnosing HF should be 200 pg/mL [13,24,25,26]. It has been suggested that, in patients with stage 4–5 CKD that exhibit symptoms of acute HF, very high levels of BNP indicate that the symptoms are related to an ischemic background. In this group of patients, a prospective study showed that, on admission, a BNP concentration >2907 pg/mL showed 71% sensitivity and 72% specificity for identifying an ischemic etiology of HF [27]. Moreover, in patients with stage 4–5 CKD, a BNP concentration >157 pg/mL was identified as an independent risk factor for a cardiovascular event; however, the sensitivity and specificity were only 65% and 56%, respectively [28]. It has also been shown that, in patients with end-stage renal failure, but without clinical symptoms of HF, the detection of BNP levels >150 pg/mL before starting renal replacement therapy was an independent risk factor for overt HF [29].

Deteriorating renal function affects the NT-proBNP concentration to a greater extent than the BNP concentration. In patients without CKD, these markers are equally important for the diagnosis of HF; however, in the context of a prognosis assessment, NT-proBNP has shown better value than BNP [1,13,26]. In patients with impaired renal function, the significance of high NT-proBNP concentrations in the diagnosis of acute HF was similar to its significance in the population of patients with eGFRs > 60 mL/min/1.73 m^2^; however, that finding was based on the adoption of cut-off thresholds that were double the typical NT-proBNP thresholds used for diagnosing HF in different age groups [13]. A standardized natriuretic peptide cut-off threshold for diagnosing chronic HF has not been clearly established to date. This uncertainty is probably due to the difficulty in determining whether the chronic increase in NT-proBNP concentration is mainly caused by damage to the myocardium or by impaired glomerular filtration [22]. Many studies have shown that an elevated NT-proBNP concentration is a negative prognostic factor in patients with CKD; however, the cut-off threshold differs significantly for different CKD severities. For example, Horri et al. conducted a study involving over 1000 patients with CKD, including 85 patients with stage 4–5 CKD. They used NT-proBNP cut-off points of 5809.0 pg/mL, for patients with eGFRs < 30 mL/min/1.73 m^2^, and 259.7 pg/mL for patients with eGFRs > 30 mL/min/1.73 m^2^ [28]. Şimşek et al. analyzed data from patients with stage 3–4 CKD. They proposed NT-proBNP cut-off points of 197 pg/mL, for predicting an increased risk of death in patients with eGFRs > 60 mL/min/1.73 m^2^, and 251 pg/mL for predicting cardiovascular death in patients with eGFRs < 60 mL/min/1.73 m^2^ [30]. That study excluded patients with end-stage kidney disease [30]. Based on the cited data, the concentration of natriuretic peptides appears to change dynamically, between the 4th and 5th stages of CKD.

NT-proBNP is also an important prognostic factor in patients on dialysis [31]. Tsai et al. showed that NT-proBNP was an indicator of intravascular fluid status [32]. The NT-proBNP concentration depended on the fluid distribution between the intra- and extracellular compartments. In patients with advanced CKD that were undergoing dialysis, NT-proBNP was a marker of fluid overload [31]. Moreover, the coexistence of high NT-proBNP concentrations and the symptoms and signs of overhydration had a synergistic effect on the risk of death and the risk of cardiovascular events [32]. However, no specific cut-off points were determined. Additionally, in patients that require repeated hemodialysis, the natriuretic peptide concentration may be influenced by the type of membrane in the dialyzer. When low-flux membranes were used for hemodialysis, the patient’s BNP concentration decreased, and the NT-proBNP concentration increased. However, when high-flux membranes were used, the patient’s BNP concentration decreased and the NT-proBNP concentration remained unchanged [33,34]. Moreover, in patients undergoing hemodialysis, the use of arteriovenous fistulas (AVFs) can be cardiotoxic, which may cause additional difficulty in interpreting the significance of the BNP concentrations. Therefore, it is difficult to interpret the significance of high natriuretic peptide concentrations in patients with advanced CKD. The cut-off thresholds can be dramatically different in different stages of the disease, and the concentrations may be affected by dialysis. This situation is similar to the situation in patients with advanced, chronic HF, where it is difficult to interpret changes in the concentration of highly sensitive T troponins, due to the many influencing factors. Thus, it remains unclear when dynamic changes in natriuretic peptide concentrations should be considered important.

## 6. Cardiotoxicity of Arteriovenous Fistulas

The implantation of a venous catheter during hemodialysis is associated with an increased rate of infection and early mortality. Hence, the principle of “fistula first” has been widely promoted. However, although AVFs are associated with better clinical outcomes compared to other forms of vascular access, they are not without potential complications [35,36]. The first reports on the potential cardiotoxicity associated with AVFs appeared over 50 years ago. Currently, the suggested management strategy is often marked with the slogan "Patient first, not fistula first, but avoid a catheter if at all possible" [37].

When an AVF is generated, it causes an immediate decrease in systemic peripheral resistance and blood leaks through the low-resistance artificial arteriovenous junction. A decrease in systemic resistance increases sympathetic activity, which increases the CO to maintain blood pressure. Therefore, the first adaptive mechanism is the occurrence of hyperkinetic circulation [37,38], which results in an increase in venous return. Gradually, the increased venous return to the ventricles (functional hypervolemia) causes an increase in the stroke volume and tends to slow the heart rate. These changes occur in every patient that receives an AVF, but the changes are generally compensatory in nature. When fistula flow (Qa) is not high and the heart remains efficient, these changes compensate for the left-to-right leak [37,39,40]. Symptoms of HF typically appear when the Qa rises to 20–50% of the CO. Chronic maintenance of an elevated CO or periodic increases in CO, in response to exercise, are dependent on the heart reserves. Exceeding the heart reserve leads to structural changes in the heart, including muscle hypertrophy, ventricle dilatation, and the gradual development of HF [37]. The minute volume capacity generated by the damaged heart becomes insufficient for the needs of the body. As a result, hypertension develops in the systemic veins and pulmonary vessels. The first signs of dysfunction are a volume overload, compensatory vasoconstriction, and an increase in systemic pressure, which further exacerbates HF. An AVF requires a satisfactory access Qa to ensure adequate dialysis. However, the paradox is that, on one hand, a low Qa value indicates fistula dysfunction and, on the other hand, a high Qa results in HF with a high CO. It is worth noting that the relationship between the Qa and CO does not appear to be linear. At the end of the 20th century, Pandeya and Lindsay presented the concept of using the Qa/CO ratio (a measure of cardiopulmonary recirculation) to monitor AVF flow in patients undergoing hemodialysis [41]. Thus, the propensity to develop symptomatic HF is believed to be proportional to the Qa, with a proposed cut-off of 2.0 L/min. As a cut-off point, this value has 89% sensitivity and 100% specificity for the diagnosis of HF in patients with a high CO [42].

The therapeutic management of HF with a high CO is difficult. Standard pharmacological treatment does not seem to be particularly effective. In recent years, several case reports showed that a surgical fistula closure was associated with improved performance in HF [43,44,45]. However, the study by Gumus and Saricaoglu deserves special attention because they analyzed 81 patients to determine potential predictors of the occurrence of right ventricular (RV) HF symptoms after an AVF insertion [46]. Prior to creating the AVF, 74% of patients presented symptoms of grades II-III chronic HF, according to the NYHA classification. Independent predictors for RV HF after an AVF were a RV longitudinal strain (RVLS) in the free wall <−19% (odds ratio (OR): 2.31, 95% CI: 1.02–3.22) and tricuspid regurgitation jet velocity (TRJV) >2.5 m/s (OR: 5.68, 95% CI: 1.21–4.38). The areas under receiver operating curves were 0.86 (95% CI: 0.55–0.89, *p* = 0.004), for RVLS, and 0.81 (95% CI: 0.55–0.89, *p* = 0.005), for TRJV > 2.61 m/s [44]. It is worth noting that the groups with and without RV HF after AVF had similar Qa values (approximately 0.5 L/min) and NT-proBNP concentrations (approximately 850 pg/mL vs. approximately 940 pg/mL). Moreover, in both groups, these values were significantly above the norm expected for patients without CKD [46]. 

Additionally, it is worth noting that, in the Gumus and Saricaoglu study, the definition of RV HF included the natriuretic peptide concentrations and a number of echocardiographic parameters (i.e., central venous pressure or right atrial pressure >16 mm Hg; tricuspid annular plane systolic excursion <16 mm, or an RV fractional area change <35% or an RV basal end- diastolic diameter >41 mm; an inferior vena cava diameter >21 mm; and <50% inferior vena cava collapsibility); the presence of significant peripheral edema, ascites, or hepatomegaly; and laboratory markers of deteriorating liver or kidney function, compared to the state before the fistula creation [46]. The literature on this subject suggests that, in patients with end-stage renal failure and concomitant NYHA I-II HF, AVF dialysis therapy can be started, but distal vessels should be used for this purpose [47]. Moreover, in patients with NYHA III HF, the decision to select AVF must be carefully considered, and it should depend on echocardiographic parameters. In patients with an LVEF < 30%, a permanent catheter placement is recommended. In patients with NYHA IV HF, a permanent catheter is elective for venous access [47]. However, due to the practical difficulties (mentioned above) in making a definitive diagnosis of chronic HF in patients with end-stage renal disease, the functional classification available does not guarantee a correct diagnosis. Indeed, many patients with advanced CKD have a normal LVEF.

Currently, we know little about how to apply the existing knowledge about the cardiotoxic potential of AVF in practice [48,49]. Among the symptoms of RV HF, it is worth noting another difficult, and yet little known, problem frequently present in patients with advanced CKD—pulmonary hypertension.

## 7. Pulmonary Hypertension in Patients with CKD

Pulmonary hypertension is defined as a mean pulmonary artery pressure ≥25 mmHg at rest, based on a direct hemodynamic measurement. Pulmonary hypertension can occur secondary to many diseases of the heart, lungs, and pulmonary vessels. Classically, there are five groups of pulmonary hypertension. The fifth group includes: hematologic disorders, systemic diseases, metabolic disorders, chronic renal failure, and disorders leading to pulmonary vascular occlusion or compression. However, for patients with advanced renal failure, including those on dialysis, the pathophysiology of pulmonary hypertension is multifactorial. The pathophysiological mechanisms include:

- overhydration
- pulmonary congestion, resulting from reduced LV compliance, and LV diastolic dysfunction (a consequence of arteriosclerosis and chronic hypertension)
- pulmonary vessel remodeling, caused by an increase in vasoactive factors (e.g., nitric oxide, prostacyclin, and endothelin)
- inflammation
- coexisting lung disease (e.g., obstructive sleep apnea and chronic obstructive pulmonary disease)
- increased CO in the course of anemia, due to the presence of an AVF [17,50]

Ultimately, pulmonary hypertension affects 21–41% of patients with chronic kidney disease and up to 60% of patients treated with hemodialysis [50,51,52,53,54,55,56]. When pulmonary hypertension is suspected, echocardiography is the first diagnostic test performed. The probability of pulmonary hypertension is established based on an evaluation of the maximum velocity of the tricuspid regurgitation velocity (Table 2), taking into account the possible coexistence of other features of RV overload.

Depending on the probability of pulmonary hypertension, assessed by echocardiography and the clinical picture, indications for cardiac catheterization are determined. Cardiac catheterization is the only examination that determines the diagnosis of pulmonary hypertension. During right heart catheterization the pulmonary capillary wedge pressure, pulmonary arterial pressure, and pulmonary vascular resistance are determined. Based on these results, three pulmonary hypertension subtypes are distinguished: (1) pre-capillary, (2) post-capillary, and (3) combined pre- and post-capillary [17].

Concurrent CKD strongly influences both the pulmonary hypertension subtype and the associated mortality. Edmonston et al. showed that, among patients without CKD, the pre-capillary subtype was dominant, and was associated with the highest risk of death. In contrast, among patients with CKD, the combined pre- and post-capillary and isolated extra-capillary subtypes dominated [17]. The multifactorial nature of pulmonary hypertension in the course of CKD suggests that the cause may be, among others, pulmonary congestion, due to reduced LV compliance and LV diastolic dysfunction [17,50]. The coincidence of CKD and pulmonary hypertension is associated with a significant increase in patient mortality; therefore, it is important to understand the pathological mechanisms underlying pulmonary hypertension. However, currently, there are many hypotheses [57,58,59,60,61], including a chronic volume overload, which can accelerate pulmonary vascular remodeling. The role of nitric oxide was also suggested because nitric oxide regulates pulmonary vascular tone, and it is a frequent target of pharmacotherapy for pulmonary arterial hypertension. Moreover, CKD adversely affects many mediators of nitric oxide metabolism (e.g., L-arginine and homocysteine). Another postulated pathological mechanism for pulmonary hypertension is an increase in fibroblast growth factor-23 (FGF-23) concentration, which is observed in the course of CKD. FGF-23 is associated with, among other things, the occurrence of LV hypertrophy and HF. Moreover, the FGF-23 concentration is correlated with pulmonary artery pressure, in some populations; however, this relationship remains unclear in patients with CKD. In addition, progressive renal dysfunction and dialysis therapy promote the activation of the inflammatory system. In this context, researchers have examined the correlation between increasing concentrations of various factors (e.g., TGF-β, IL-6, or IL-10) and the presence of pulmonary hypertension [57,58,59,60,61]. Ultimately, despite the high prevalence and increased risk of mortality associated with pulmonary hypertension in patients with CKD, pulmonary hypertension remains insufficiently understood in patients with CKD [61].

## 8. Whether CKD Affects Basic Pharmacotherapy of HF?

The main topic of our review is the issue of diagnostic difficulties, but it is impossible not to refer to the issue of pharmacotherapy. Medications that can improve prognosis in HF with reduced LVEF are angiotensin-converting enzyme inhibitors (ACE), angiotensin II receptor blockers (ARB), angiotensin receptor neprilysin inhibitors (ARNI), b-blockers, and mineralocorticoid receptor antagonists (MRA). Recently sodium-glucose cotransporter 2 inhibitors (SGLT-2) have joined this group, and according to current ESC guidelines blockade of the renin-angiotensin-aldosterone system with ACE, ARNI or ARB, beta-blocker, SGLT-2 inhibitor and MRA should be started as soon as possible after the diagnosis of HF with reduced LVEF [1]. Until recently, it was considered that the coexistence of CKD did not affect the general principles of pharmacological management in HF [22]. However, the scientific evidence for the efficacy of conventional treatment of HF with decreased LVEF is lower the more advanced CKD is. The strongest evidence is for beta-blocker. The studies on bisoprolol, carvedilol and metoprolol have also shown an improvement in the prognosis of patients with HF in the case of concomitant CKD [62,63,64]. Although ACE and ARB can cause eGFR to decrease in patients with HF, the benefit of angiotensin blockade in terms of prognosis in patients with HF and reduced LVEF seems to be maintained [65,66]. Evidence for the efficacy of MRA in the treatment of patients with HF and advanced CKD are limited [67,68]. For decades another serious challenge was the management of HF with preserved LVEF, for which there was no treatment improving prognosis [22,69]. This situation changed with the publication of the results of the EMPEROR-Preserved trail. Empagliflozin is the first molecule to improve the prognosis of HF patients with preserved LVEF [70]. Considering the data on the nephroprotective effect of SGLT-2 inhibitors, and the prevalence of HF with preserved EF among CKD patients, the results of this study are particularly noteworthy [71,72]. However, a detailed discussion of the principles and doubts regarding the pharmacotherapy of HF in the coexistence of advanced CKD is beyond the scope of this review. 

## 9. Conclusions

Among patients that first develop chronic progressive renal failure, it may be difficult to confirm concurrent HF. The clinical symptoms of HF and advanced CKD may be confusingly similar, particularly in patients on dialysis. The eGFR has a significant impact on natriuretic peptide concentrations—indeed, increases in these peptides result from both damage to the heart and their impaired elimination in the kidneys. Definitive cut-off points for BNP and/or NT-proBNP concentrations have not been established for diagnosing HF in patients with CKD. However, elevated levels of these peptides have been shown to have negative prognostic significance. Therefore, for patients with intermediate or preserved LVEF and CKD, there remains a need for new diagnostic criteria that can confirm or exclude HF as the primary cause of fluid overload. Moreover, the correct diagnosis of HF in patients classified as pre-dialysis may be a key issue in selecting the optimal vascular access. Despite the convincing pathophysiological basis, little evidence is available to support the potential cardiotoxicity of fistulas. However, new research has indicated that it may be possible to use an echocardiographic assessment of RV strain.

## Figures and Tables

**Table 1:** Benefits and limitations of additional study methods for evaluating overhydration.

Study Methods	Benefits	Limitations
**Electrical bioimpedance**	The method has been validated against the “gold standard” Recommended method Assessment of total body hydration Non-invasive examination Simple execution Repeatability of measurements Low cost Short waiting time for the result Performed “at the bedside” Detection of subclinical forms of fluid overload	Numerous factors influence measurement accuracy Parameters are assessed based on mathematical estimates of data from the population of healthy Caucasian people It provides only a summary assessment of total body fluid overload Limited sensitivity of fluid assessment in the so-called third space
**Ultrasound evaluation of the inferior vena cava**	Low cost Performed “at the bedside” Non-invasive Possibility of multiple inspections Results available immediately after the end of the test	▪ Assesses blood volume only (intravascular fluid) ▪ Requires cooperation from the patient ▪ The abnormalities found may not be specific to overhydration alone ▪ Possibility of multiple inspections
**Lung Ultrasound**	▪ A simple, repeatable method ▪ Favorable learning curve ▪ Low cost ▪ Wide availability ▪ Low hardware requirements: any ultrasound head, use of portable devices ▪ Preformed “at the bedside” ▪ Non-invasive ▪ Detection of subclinical forms of pulmonary congestion ▪ Possible to make accurate assessments of pleural cavities ▪ Results are available immediately after the end of the test	▪ Requires differentiation of the causes of B-line artifacts, based on clinical data ▪ Difficult to assess several things, including: the presence of a large amount of fluid in the pleural cavities; pneumonia; interstitial lung diseases

**Table 2:** Probability of pulmonary hypertension (PH), stratified by echocardiography results.

Maximum Velocity of the Tricuspid Regurgitation Velocity	Probability of PH
≤2.8 m/s (TVPG ≤ 31 mm Hg)	Low
2.9–3.4 m/s (TVPG 32–46 mm Hg)	Intermediate
>3.4 m/s	High

TVPG: tricuspid valve pressure gradient.

## Data Availability

Not applicable.
